# Incidence of postural hypotension recorded in UK general practice: an electronic health records study

**DOI:** 10.3399/BJGP.2022.0111

**Published:** 2022-10-18

**Authors:** Cini Bhanu, Irene Petersen, Mine Orlu, Daniel Davis, Kate Walters

**Affiliations:** Research Department of Primary Care and Population Health, University College London, London.; Research Department of Primary Care and Population Health, University College London, London.; UCL School of Pharmacy, University College London, London.; MRC Unit for Lifelong Health & Ageing, University College London, London.; Research Department of Primary Care and Population Health, University College London, London.

**Keywords:** hypotension, orthostatic, general practice, postural hypotension, primary health care

## Abstract

**Background:**

Postural hypotension is a common condition associated with adverse outcomes in older adults. General practice plays an important role in identification of the condition.

**Aim:**

To examine the incidence of postural hypotension between 2008 and 2018 in general practice and how trends vary by age, sex, year, and social deprivation.

**Design and setting:**

Retrospective cohort study using electronic health records from the IQVIA Medical Research Data (IMRD) between 2008 and 2018.

**Method:**

Patients were included if they were aged ≥50 years. Incident postural hypotension was identified as a new (first) recording of a postural hypotension code. Recording of incident postural hypotension was estimated per 10 000 person–years at risk (PYAR) according to age, sex, year, and social deprivation. Incident rate ratios were estimated by multivariable Poisson regression.

**Results:**

Of 2 911 260 patients, 24 973 had an electronic record indicating a new diagnosis of postural hypotension between 2008 and 2018. This was equivalent to 17.9 cases per 10 000 PYAR in males (95% confidence interval [CI] = 17.6 to 18.2) and 16.2 cases per 10 000 PYAR in females (95% CI = 15.9 to 16.5). A significant age–sex interaction was identified. Recorded postural hypotension rate increased with age and social deprivation, and reduced between 2008 and 2018. The rate was higher in males compared with females, particularly in older age groups (>80 years).

**Conclusion:**

To the authors’ knowledge, this is the first study to quantify incident recorded postural hypotension in general practice. The rate is lower than expected compared with studies in screened older populations. Potential barriers to identification include underreporting, underdetection owing to lack of time and/or poorly standardised methods of measurement, and poor coding. Future research should investigate current practice and approaches for increased detection such as education, practical methods of screening, and standardised measurement of postural blood pressure.

## INTRODUCTION

Postural (orthostatic) hypotension is a common, yet frequently overlooked, condition associated with serious adverse outcomes in later life.^1^ It is estimated to affect around 20% of community-dwelling older adults^2^^,^^3^ and between 20% and 31% of those living in long-term care.^2^^,^^3^ In the UK, the reported prevalence of postural hypotension has ranged from 28% in older females,^4^ up to 81% of older adults screened using continuous blood pressure (BP) monitoring.^5^

Postural hypotension is usually defined as a reduction in systolic BP of ≥20 mm Hg or diastolic BP of ≥10 mm Hg within 3 min of assuming an erect posture or head-up tilt to at least 60 degrees on a tilt table.^6^ Its resulting effect on reduced cerebral blood flow is associated with falls, fractures, ischaemic events, cognitive impairment, and increased mortality.^3^^,^^7^ Older people with postural hypotension are 2.5 times more likely to have recurrent falls, compared with those without.^8^ Falls are estimated to cost the NHS more than £2.3 billion per year, including acute care for fractures and social care.^9^

Early detection in patients who are symptomatic or in those with certain risk factors may prevent some of these complications. General practice plays an important role in identification; however, current guidelines for detecting postural hypotension are varied and based on limited evidence.^10^ In the UK, screening is recommended for older adults presenting after a fall or in people with hypertension who are symptomatic, have diabetes, or aged >80 years.^9^^,^^11^ US guidelines recommend that postural BP is checked in high-risk groups.^1^ A large proportion of people with postural hypotension are asymptomatic, and are therefore likely to remain undetected unless screened because they are in high- risk groups.^2^

To the authors’ knowledge, no studies have examined the incidence of patients with postural hypotension presenting to general practice, and it is unclear how well GPs identify symptomatic postural hypotension in normal practice and whether this varies in different population subgroups. This study aims to examine:

- the incidence of recorded postural hypotension over the past decade in general practice electronic health records; and
- how trends in incidence of recorded postural hypotension vary by age, sex, and sociodemographic characteristics.

## METHOD

### Design

This was a retrospective cohort study using routinely collected healthcare data.

**Table:** How this fits in

Postural hypotension is a common, yet frequently overlooked condition associated with serious adverse outcomes in older people. Timely identification in general practice may reduce the onset of adverse sequelae. This study found that recording of postural hypotension in electronic GP records is low and poorly reflective of expected rates in the community. These findings suggest there are barriers to identification and recording of postural hypotension in general practice, indicating potential for standardised methods of detection and screening.

### Data source

This study used data from anonymised electronic primary care records contributing to the IQVIA Medical Research Data (IMRD) that includes over 18 million patients^12^ from over 700 practices. These are broadly representative of UK practices in terms of age, sex, practice size, geographical distribution, and sociodemographic characteristics.^13^ GPs systematically recorded medical diagnoses and symptoms using the Read classification coding system during routine health care.^14^ This includes data from consultations with clinicians (GPs and nurses) and data (for example, diagnoses and health measurements) coded into healthcare records from letters received from secondary care (for example, hospital admissions and out-patient clinics). Social deprivation is measured using linked population census data on the Townsend score (based on postcode sector area of residence, owner-occupation, car ownership, overcrowding, and unemployment). This is split into Townsend quintiles 1–5 (1 being the least deprived).^15^

In the UK, health care is free to access, and individuals typically register with a GP in their local area. Approximately 98% of the UK population are registered with a GP^16^ and over 90% of NHS contacts are in general practice.^17^

### Study population

The source population was all patients aged ≥50 years, registered with a GP practice contributing data to the IMRD at acceptable quality and mortality reporting levels,^18^^,^^19^ for at least 1 year between 1 January 2000 and 31 December 2018.

### Measurement of outcome

Cases of postural hypotension were identified in patients who had a new (first) record of a Read code for postural hypotension between 1 January 2000 and 31 December 2018, at least 6 months after they registered with the GP. A list of all diagnosis codes was constructed using established methods.^20^ In this study a specific code list was used that included four Read codes with high certainty of a validated diagnosis:

- ‘O/E — BP reading: postural drop’ (medcode 2468.00);
- ‘orthostatic hypotension’ (G870.00);
- ‘postural hypotension’ (G870.11); and
- ‘Parkinsonism with orthostatic hypotension’ (F130300).

The number of individuals with a newly recorded diagnosis was determined by age (in 10-year age bands), sex, year, and quintiles of Townsend score.

### Statistical analysis

The recording of coded postural hypotension was estimated per 10 000 person–years at risk (PYAR) for individuals who were registered at some point between 2009 and 2018. Incidence rates of recorded postural hypotension were reported per 10 000 PYAR with 95% confidence intervals (95% CIs) for males and females overall, for age bands, Townsend quintile, calendar year, and stratified by sex. Annual rates were graphed to examine the time trends. Incidence rate ratios were estimated by multivariable Poisson regression and estimates were mutually adjusted by sex, age, year, and social deprivation. Models were run with and without interaction terms and the likelihood ratio test was performed to analyse which model fit best. This identified a significant age–sex interaction. Therefore, all results are presented stratified by sex. Analyses were carried out using Stata (version 16.0).

### Patient and public involvement

A patient and public involvement (PPI) advisory group was consulted throughout this study. This included three older members (aged >65 years) who either had experience of postural hypotension themselves or cared for an older adult who has experienced postural hypotension. They contributed to the interpretation of the results and recommendations for clinical practice.

## RESULTS

In total, 24 973 individuals (among 2 911 260 patients) had an electronic record indicating a new diagnosis of postural hypotension between 2008 and 2018. This was equivalent to 17.9 cases per 10 000 PYAR in males (95% CI = 17.6 to 18.2) and 16.2 cases per 10 000 PYAR in females (95% CI = 15.9 to 16.5). A significant age–sex interaction was found. Therefore, all results are presented stratified by sex (Table 1).

**Table 1.** Incidence rates of recorded postural hypotension per 10 000 PYAR (95% CI) for males and females overall, for age bands, Townsend quintile, calendar year, and stratified by sex

**Characteristic**	**Rate per 10 000 PYAR (95% CI)**		**Adjusted^a^ IRR (95% CI)**	

	**Males**	**Females**	**Males**	**Females**
**Overall**	17.9 (17.6 to 18.2)	16.2 (15.9 to 16.5)	—	—

**Age band, years**				
50–69	4.4 (4.1 to 4.7)	3.9 (3.6 to 4.1)	1	1
60–69	11.6 (11.1 to 12.1)	8.4 (8.0 to 8.8)	2.7 (2.5 to 2.9)	2.2 (2.0 to 2.4)
70–79	30.9 (30.0 to 31.9)	23.3 (22.5 to 24.1)	7.1 (6.7 to 7.6)	6.0 (5.6 to 6.5)
80–89	62.5 (60.4 to 64.6)	50.7 (49.2 to 52.3)	14.4 (13.4 to 15.4)	12.9 (12.1 to 13.9)
≥90	82.9 (76.4 to 89.7)	54.1 (50.9 to 57.6)	19.0 (17.2 to 21.0)	13.8 (12.6 to 15.1)

**Townsend quintile**				
1	16.6 (16.1 to 17.3)	14.3 (13.8 to 14.8)	1	1
2	16.9 (16.2 to 17.5)	14.1 (13.5 to 14.7)	0.9 (0.9 to 1.0)	0.9 (0.9 to 1.0)
3	17.9 (17.2 to 18.7)	15.8 (15.1 to 16.4)	1.1 (1.0 to 1.2)	1.0 (1.0 to 1.1)
4	18.9 (18.1 to 19.7)	19.0 (18.3 to 19.8)	1.2 (1.1 to 1.2)	1.2 (1.1 to 1.3)
5	21.7 (20.6 to 22.8)	22.2 (21.1 to 23.3)	1.4 (1.3 to 1.5)	1.4 (1.4 to 1.5)

**Year**				
2009	19.3 (18.3 to 20.3)	18.8 (17.9 to 19.7)	1	1
2010	18.5 (17.6 to 19.5)	17.9 (17.0 to 18.8)	1.0 (0.9 to 1.0)	1.0 (0.9 to 1.0)
2011	19.1 (18.1 to 20.1)	16.7 (15.8 to 17.6)	1.0 (0.9 to 1.1)	0.9 (0.8 to 1.0)
2012	17.9 (16.9 to 18.8)	17.0 (16.1 to 17.9)	0.9 (0.9 to 1.0)	0.9 (0.9 to 1.0)
2013	18.5 (17.5 to 19.5)	16.6 (15.8 to 17.5)	1.0 (0.9 to 1.0)	0.9 (0.8 to 1.0)
2014	17.7 (16.8 to 18.7)	15.6 (14.8 to 16.5)	0.9 (0.8 to 1.0)	0.9 (0.8 to 0.9)
2015	17.4 (16.4 to 18.5)	14.1 (13.2 to 15.0)	0.9 (0.8 to 1.0)	0.8 (0.7 to 0.8)
2016	16.2 (15.1 to 17.4)	14.7 (13.7 to 15.8)	0.8 (0.8 to 0.9)	0.8 (0.8 to 0.9)
2017	15.7 (14.5 to 16.9)	13.8 (12.8 to 14.9)	0.8 (0.7 to 0.9)	0.8 (0.7 to 0.8)
2018	16.0 (14.8 to 17.3)	13.5 (12.4 to 14.6)	0.8 (0.7 to 0.9)	0.7 (0.7 to 0.8)

a *From multilevel Poisson regression adjusted by age band, Townsend quintile, and year, and stratified by sex. IRR = incidence rate ratio. PYAR = person–years at risk.*

**Figure 1.**
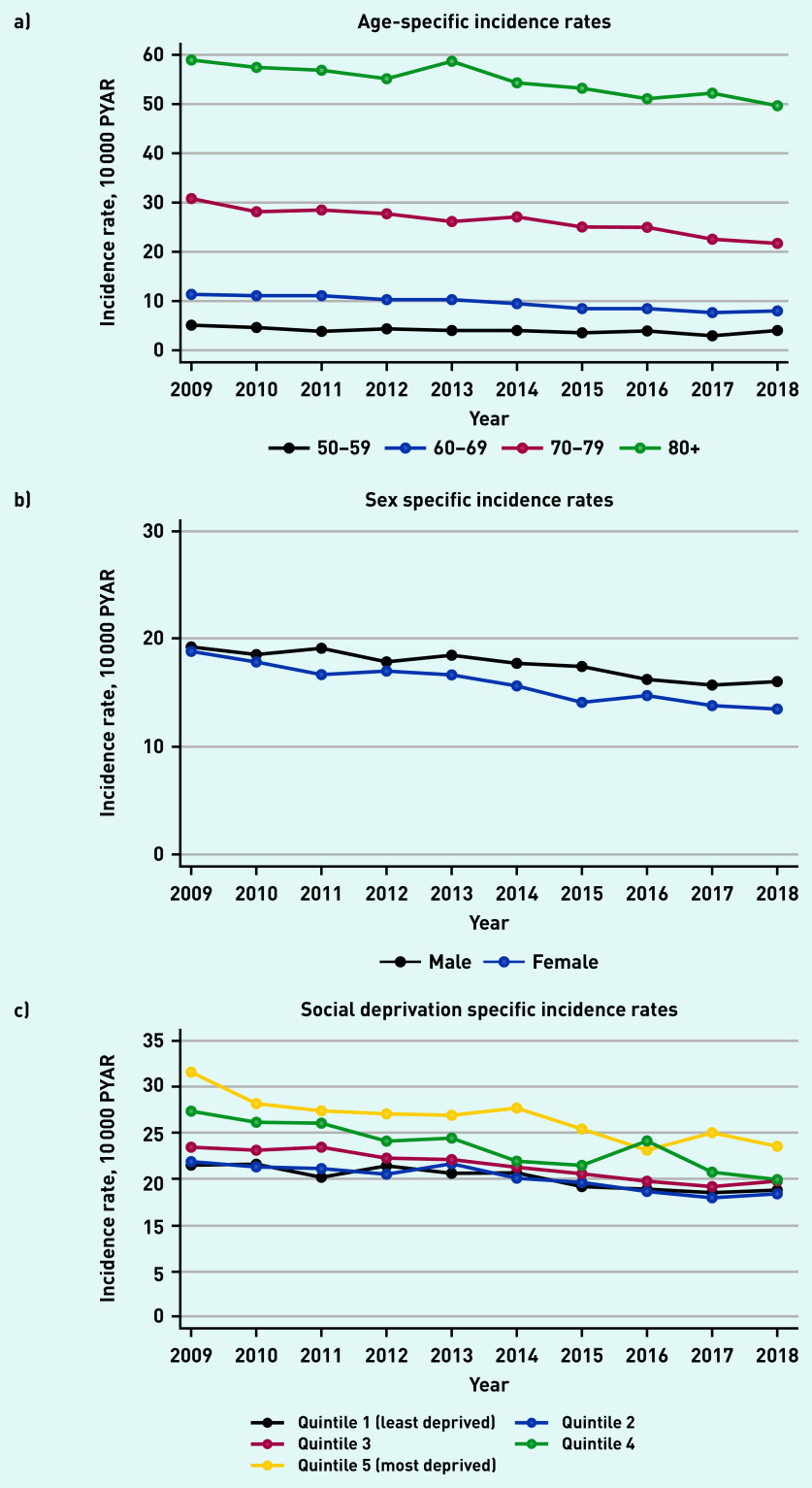
*Incidence of recorded new GP diagnosis of postural hypotension from 2009 to 2018. a) Age-specific incident rates; b) sex-specific incidence rates; and c) social deprivation-specific incidence rates. PYAR = person–years at risk.*

There were differences in trends by age, sex, Townsend deprivation quintile, and year (Table 1 and Figure 1). The incidence of postural hypotension increased significantly with age to an adjusted incidence rate ratio (IRR) of 19.0 (95% CI = 17.2 to 21.0) in males in the oldest age group (≥90 years), compared with males aged 50–69 years. In females aged ≥90 years, the adjusted IRR was 13.8 (95% CI = 12.6 to 15.1), compared with females aged 50–69 years.

For patients in the most socially deprived Townsend quintile 5, the adjusted IRR in males was 1.4 (95% CI = 1.3 to 1.5) and in females was 1.4 (95% CI = 1.4 to 1.5), compared with the least deprived Townsend quintile. Time trends show a small but significant reduction over the years. In 2018, the adjusted IRR in males was 0.8 (95% CI = 0.7 to 0.9) and 0.7 (95% CI = 0.7 to 0.8) in females, compared with 2009 (Table 1).

**Figure 2.**
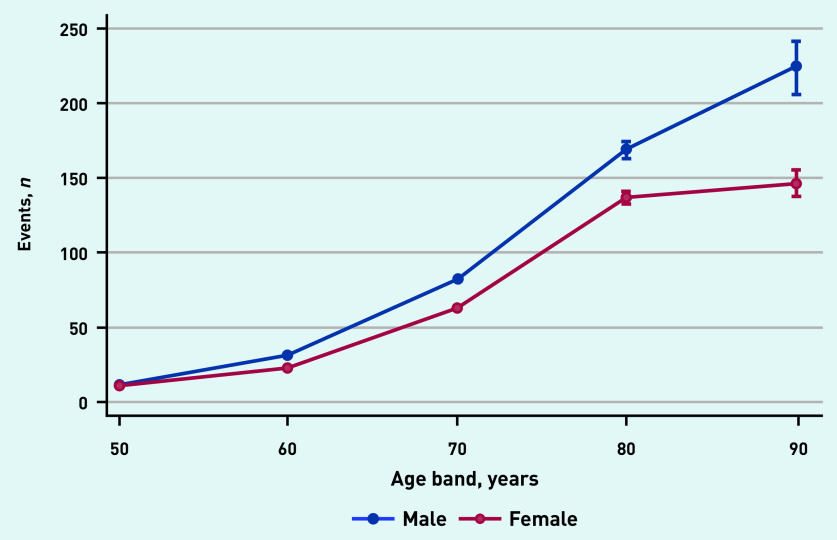
*GP recording of postural hypotension by age and sex.*

A significant age–sex interaction was identified. The incidence of recorded postural hypotension increased at a greater rate by age band among males, compared with females (Figure 2).

## DISCUSSION

### Summary

To the authors’ knowledge, this is the first study to quantify recorded diagnoses of postural hypotension among patients in general practice.

The rate of recorded postural hypotension diagnoses in primary care among males aged ≥50 years was 17.9 cases per 10 000 PYAR (95% CI = 17.6 to 18.2) and 16.2 cases per 10 000 PYAR in females (95% CI = 15.9 to 16.5). This rate is much lower than expected from studies in screened older populations that estimates the prevalence of postural hypotension in community-dwelling adults to be 22%, and 23.9% in long-term care.^3^

The rate of recorded postural hypotension increased substantially with age as anticipated; increased with greater levels of social deprivation; reduced slightly over time between 2008 and 2018; and was higher in males compared with females, particularly in older age groups (>80 years).

### Strengths and limitations

The main strength of this study is the large population sample (just under 3 million patients) enabling precise estimates of rates of case recording in primary care. The IMRD is also broadly demographically representative of patients in UK primary care. It was not possible to examine rates of postural hypotension by ethnic group in this study because of the high levels of missing data and the IMRD generally under-represents groups from minority ethnic backgrounds.

**Figure 3.**
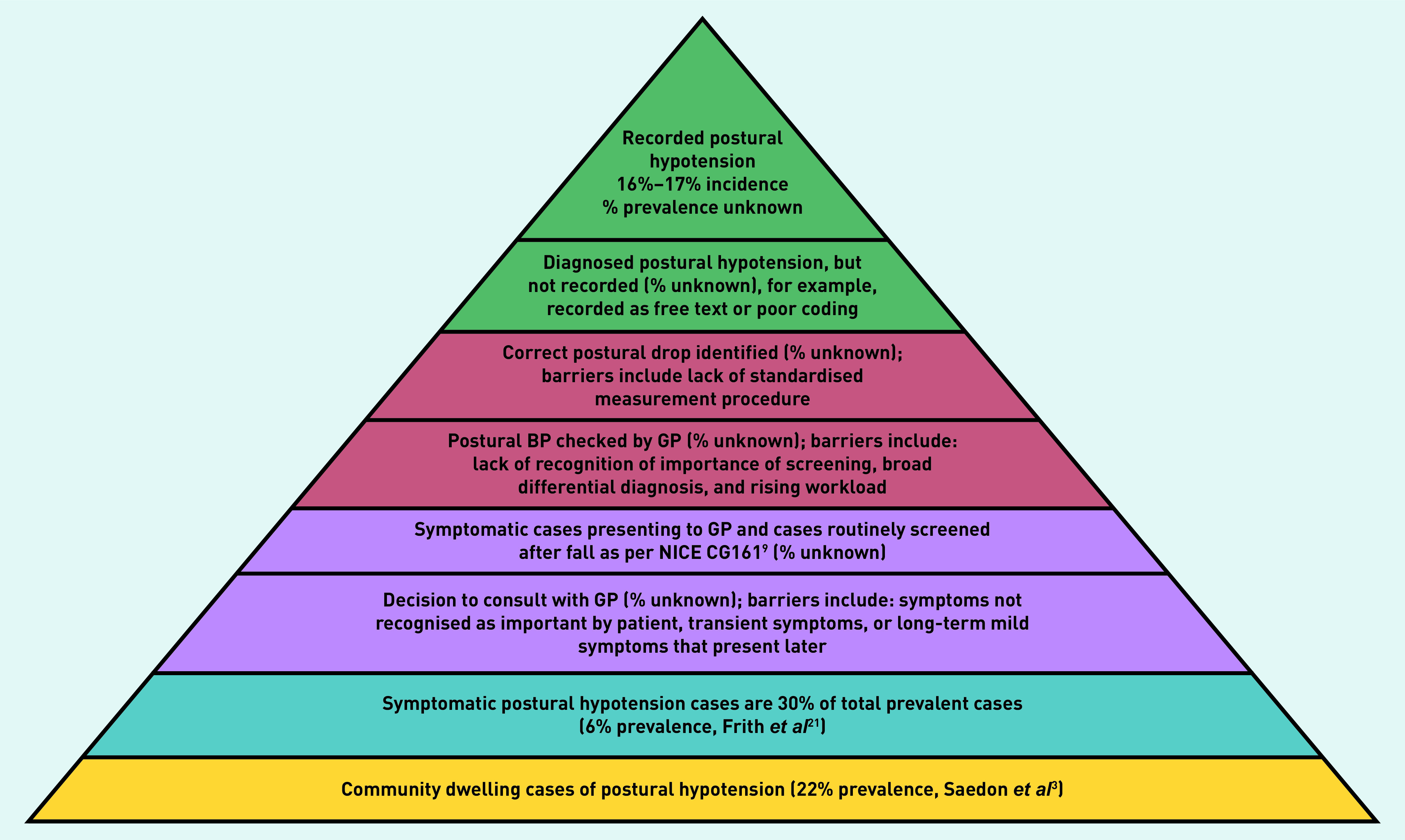
*Barriers to recording of postural hypotension cases in UK general practice. BP = blood pressure. NICE = National Institute for Health and Care Excellence.*

There are, however, limitations in estimating the community incidence of postural hypotension from dynamic, longitudinal GP records. In this study, cases were defined with a high specificity diagnostic list of Read codes as the authors were interested in GP-recorded postural hypotension cases specifically. However, there are several barriers that likely resulted in lower recording of coded postural hypotension in the electronic GP records, compared with community numbers (Figure 3).

Barriers include patient underreporting to GPs. This is likely because of people with asymptomatic cases not presenting to primary care (only an estimated 30% of cases present with the classical symptoms of dizziness or light-headedness)^21^ and because of a lack of routine postural BP screening in general practice. A further smaller percentage of patients may present with non-specific symptoms^21^ such as intermittent blurred vision, which the PPI group agreed were less likely to trigger presentation to their GP.

Further factors include underdetection by clinicians in primary care, which might be because of: limited recognition of postural hypotension in clinical guidelines and its significance; atypical symptoms and a broad differential diagnosis; rising workloads; lack of time to screen; and poorly standardised methods of postural BP measurement leading to fewer diagnoses. Finally, there is likely poor or inconsistent coding of postural hypotension in electronic records because of variable use of appropriate code terms, use of free-text BP recording, and symptom codes. For cases that are coded, it is not possible to be certain of the validity of GP recording (for example, whether the Freeman consensus definitions are being used).^6^

Nevertheless, the rate of recorded postural hypotension identified in this study represents a clinically meaningful group who are likely to have a clinical diagnosis. The patients identified are likely to be those with the most severe postural hypotension, representing individuals who are symptomatic presenting to their GP or those identified following a fall where a postural BP was screened for (as advised by guidelines).^8^ This is a key group of patients, providing new insight into the identification of postural hypotension in general practice to aid further understanding of its significance.

### Comparison with existing literature

To the authors’ knowledge, there are no studies examining the incidence of postural hypotension among community-dwelling older adults or in primary care to make direct comparisons. It is difficult, therefore, to evaluate the extent of underdiagnosis of incident postural hypotension in primary care. A recent systematic review and meta-analysis found the pooled prevalence of postural hypotension to be 19% for 23 screened primary care cohorts.^2^ A further systematic review on epidemiological studies in community-dwelling older adults found that the prevalence of orthostatic hypotension in screened populations (including, therefore, both asymptomatic and symptomatic orthostatic hypotension) was 22%, and 23.9% for those in long-term care.^3^ It is also estimated to affect 30% of older people with diabetes.^22^

The higher incidence of recorded postural hypotension diagnoses among males compared with females, and the significant age–sex interaction, likely reflects the known similarities in underlying pathology between postural hypotension and cardiovascular disease (CVD), which is well-established to affect males to a greater extent.^10^

The steep increase in incidence of recorded postural hypotension by increasing age band was expected and consistent with knowledge about the aetiology of this condition.^2^^,^^3^^,^^10^ Physiological changes associated with the natural ageing process causing reduced baroreceptor sensitivity and altered cardiovascular functions increases susceptibility to postural hypotension.^8^

The study found rising rates of recorded postural hypotension in groups with greater social deprivation. This may be because of a greater prevalence of polypharmacy, comorbidity, and CVD among this population, as previously described in the literature.^23^

The finding in the current study that the rate of recorded postural hypotension followed a slight downward trend from 2008 to 2018 may reflect evidenced changes in rising GP workload during this period and reducing priority of postural hypotension detection among other chronic disease management and GP work.^24^ Between 2007 and 2014, the overall workload of GPs in England rose by 16%.^24^

### Implications for research and practice

To the authors’ knowledge, this study provides the first insight into current practice and identification of postural hypotension in routine general practice, assimilating data and trends over a 10-year period.

Standardised recording of postural BP may help increase identification and recording of postural hypotension in GP records. Gibbon and Frith suggest that a postural BP drop detected within 60 s of standing upright is adequate and more likely to be associated with adverse clinical outcomes.^1^ This is a pragmatic approach that could be incorporated into existing routine care for high-risk groups including older adults (such as the NHS ‘Over 75 health check’), and can be carried out by auxiliary healthcare professionals or via ambulatory home BP monitoring, which is now more widely used.^25^

Early identification of postural hypotension (that is, before the onset of clinical sequelae such as falls and ischaemic events) may allow for a window of opportunity. This can be used to adjust high-risk drugs, optimise CVD status, and provide practical advice on hydration that may reduce subsequent adverse outcomes.

Finally, postural hypotension and its association with serious adverse outcomes in older people is gaining attention in research. It is recognised as an important marker of neurovascular dysfunction and a contributor to cognitive decline.^7^ Therefore, understanding current practice and approaches for improving postural hypotension detection is increasingly important.

Future research should consider age–sex interactions, with greater differences in rates of postural hypotension among males and females in older age groups (>70 years). This study provides context for future research to investigate the potential benefits of routine screening of postural BP in general practice among high-risk patients.

## Data

The authors are able to provide the Stata do-files on request (not the data itself).
